# Se préparer face aux épidémies Une stratégie de renforcement des compétences en sciences sociales en Afrique

**DOI:** 10.48327/mtsi.v3i4.2023.440

**Published:** 2023-10-30

**Authors:** Alice DESCLAUX, Blandine BILA, Marc EGROT, Khoudia SOW

**Affiliations:** 1 TransVIHMI, Université de Montpellier, IRD, INSERM, Montpellier, France; 2 Institut de recherche en sciences de la santé, Ouagadougou, Burkina Faso; 3 LPED (Laboratoire Population Environnement Développement), IRD, Université d'Aix-Marseille, Marseille, France; 4 CRCF (Centre régional de recherche et de formation à la prise en charge du VIH et des maladies associées de Fann), Dakar, Sénégal

**Keywords:** Épidémies, Sciences sociales, Formation, Interdisciplinarité, Préparation, Afrique de l'Ouest, Epidemics, Social sciences, Capacity building, Interdisciplinarity, Preparedness, West Africa

## Abstract

La pandémie de Covid a rappelé la nécessité de se préparer aux épidémies et pandémies et de prendre en compte leurs dimensions sociopolitiques en développant des approches socio-anthropologiques et interdisciplinaires. Après la crise, le défi est celui de l'opérationnalité. Comment rendre davantage visibles ces dimensions? Comment faire connaître des analyses qui peuvent aider à humaniser les réponses institutionnelles, rendre visibles les inégalités pour les limiter, mettre au jour les déterminants structurels de la transmission, et définir des interventions efficaces, scientifiquement fondées, éthiquement justes, et respectueuses de la diversité? Comment assurer une collaboration entre des chercheurs et des intervenants de la réponse aux épidémies sur une base de connaissances partagées?

Cet article présente une initiative de renforcement de capacités développée en Afrique francophone par le Réseau Anthropologie des Épidémies Émergentes (RAEE), montée avec d'autres réseaux (Sonar-Global) et institutions (CRCF, IRD). Il décrit et analyse un dispositif qui associe une méthode de travail, des contenus scientifiques et des outils didactiques. Deux publics sont visés: celui des chercheurs en sciences sociales (niveau Master et plus) et celui d'acteurs socio-sanitaires opérationnels (santé publique, santé communautaire, ONG, travailleurs sociaux). Les contenus en termes de compétences à acquérir (savoirs, savoir-faire) sont précisés. Les connaissances à transmettre sont organisées en 13 modules: l'introduction; le cadre de la réponse aux épidémies; l’émergence et le One Health; la résistance aux antimicrobiens; le risque infectieux (inégalités, stigmatisation et prévention); les savoirs (circulations et interprétations); les services de santé (lieux de risque et de soins); les mesures de santé publique (enfermement et distanciation); les expériences (souffrance des malades et mobilisations); la mort (sens et rituels); la vaccination (innovation, équité et hésitation); les cycles épidémiques (préparation, réponse et rétablissement); les défis, les méthodes, l’éthique et la gouvernance; et la conclusion.

Les premières formations réalisées au Sénégal et au Burkina Faso auprès d'universitaires enseignants-chercheurs et jeunes chercheurs d'Afrique et de France ont été évaluées positivement par les participants. Ils estiment avoir acquis des connaissances en sciences sociales des épidémies mais aussi en santé publique, ce qui leur a donné les bases nécessaires pour communiquer et développer des collaborations (en recherche et en intervention) avec des acteurs socio-sanitaires. Le dispositif semble pouvoir être étendu au travers de nouvelles sessions de formation à l'initiative d'autres institutions, qui pourront utiliser le manuel.

## Abstract (en)

The Covid pandemic was a reminder of the need to be prepared for epidemics and pandemics and to take into account their socio-political dimensions by developing socioanthropological and interdisciplinary approaches. In the post-crisis era, the challenge is one of operationality. How can these dimensions be made more visible? How can we develop analyses that can help to humanize institutional responses, make inequalities visible to limit them during the crisis, reveal structural determinants of transmission, and define interventions that are scientifically sound, ethically just and respectful of diversity?

Three strategies are relevant to meet these challenges: (1) more social scientists in Frenchspeaking Africa must get expertise on epidemics to investigate associated issues before, during and after epidemic crisis; (2) public health professionals, health and social workers must get informed about social, historical, economic and political aspects of epidemics that shape risk, care and control; (3) collaborations between researchers and those involved in responding to epidemics on the basis of shared knowledge must develop.

This article presents a capacity-building initiative developed in French-speaking West Africa by the Anthropology of Emerging Epidemics Network (RAAE), in conjunction with other networks (Sonar-Global) and institutions (CRCF, IRD). It describes and analyzes a program that combines a working method, a scientific content and teaching tools. This program benefited from previous training experiences and gathered expertise from about 25 social scientists, mainly medical anthropologists, who have worked on various epidemics and pandemics such as AIDS, Ebola, plague, Covid and dengue in West Africa and beyond. The process to develop the course was based on workshops followed by redaction periods, then testings for content and tools during training sessions.

The course focuses on two audiences: social science researchers (with a Master degree level and above) and social and health workers (public health, community health, NGOs, social workers). For the former, the course aims at reinforcing theoretical and methodological skills through the presentation of issues, key concepts, selected theoretical developments, themes and bibliographical references. For the latter, the course is based on modules about operational issues that can be taken separately, to better adapt the content of trainings to local teams’ needs. For both, a glossary includes 100 definitions of public health, medical and social science vocabulary, relevant to epidemics. The content in terms of skills to be acquired (knowledge, know-how) is presented briefly in the article. Both the scientific content and learning methods and tools are presented in a manual (Desclaux et al., Anthropologie appliquée aux épidémies émergentes, 2022 [5]) as well as on Sonar-Global (English) and RAEE (French) websites (www.sonar-global.eu, www.raee.fr).

The knowledge to be imparted is organized into 13 modules: introduction; the framework for responding to epidemics; emergence and One Health; antimicrobial resistance; infectious risk (inequalities, stigmatization and prevention); knowledge (circulation and interpretation); health services (places of risk and care); public health measures (lockdown and distancing); experiences (suffering of the sick and mobilization); death (meaning and rituals); vaccination (innovation, equity and hesitation); epidemic cycles (preparation, response and recovery); challenges, methods, ethics and governance; and conclusion.

The first training courses held in Senegal and Burkina Faso for university researchers and young scholars from Africa and France were positively evaluated by the participants. They report that they have acquired knowledge in epidemic social science, but also in public health, which has given them the necessary basis for communicating and developing collaborations (in research and intervention) with social and health actors. The model could be duplicated with adaptation for new training sessions organized by other institutions, for which a manual is available.

## Introduction

Face à la menace d’épidémies de plus en plus fréquentes, la « préparation » est désormais sur l'agenda des institutions de santé publique nationales et globales, comme des États [11]. Mais cette question n'est plus abordée comme elle l’était en 2019, avant la pandémie de Covid. La pandémie a suscité une prise de conscience partagée à plusieurs niveaux, concernant notamment la nécessité de considérer en amont les contextes et mécanismes des émergences, puis le caractère central des aspects sociopolitiques dans la réponse, et de recourir à des approches multidisciplinaires dans la recherche comme dans l'intervention [19].

L'approche des deux types d’émergence habituellement distingués a été étoffée. Les zoonoses font désormais l'objet de vastes programmes de recherche et de prévention et l'approche One Health (Une seule santé) est entrée dans les savoirs et dans les institutions au travers notamment de plateformes qui rassemblent des experts en santé humaine, animale et environnementale [10]. La résistance aux antimicrobiens (notamment l'antibiorésistance) est désormais considérée comme une « pandémie silencieuse » [14] de plus en plus documentée. Le rôle des aspects sociopolitiques dans l'exposition au risque infectieux, de même que les effets sociétaux de la gouvernance de la « réponse » (en particulier des mesures de santé publique) et l'impact de l'infodémie, ont été démultipliés par la dimension globale de l’épidémie [15]. Il est maintenant établi que la « communication de risque » définie au niveau global et l'approche comportementale ne sont pas suffisantes: elles doivent être complétées par une connaissance des contextes socioculturels et politiques et par une évaluation critique des stratégies de santé publique [1, 2]. La nécessité de s'appuyer sur des recherches en sciences sociales et des approches multidisciplinaires, déjà affirmée au sortir de l’épidémie d’Ébola en Afrique de l'Ouest (2013-2016), a été encore une fois proclamée [3, 7, 13, 16, 17, 21]. Après l'expérience de trois années de pandémie, le défi principal est maintenant celui de l'opérationnalité.

Comment organiser l'interdisciplinarité tout en articulant recherche fondamentale et opérationnalité, pour aborder les enjeux sociopolitiques des épidémies à hauteur de leur importance sur le terrain? Comment l'appliquer aux champs thématiques des émergences liés aux zoonoses (« une seule santé ») et à la résistance aux antimicrobiens? Plus spécifiquement, comment assurer que des chercheurs et des intervenants de la réponse aux épidémies puissent collaborer sur une base de connaissances partagées? Concrètement, quel dispositif de renforcement des capacités peut permettre d'avancer sur ces questions et contribuer ainsi à la préparation face aux épidémies? Cet article présente une initiative développée en Afrique francophone.

## Le Réseau Anthropologie des Épidémies Émergentes

Le Réseau Anthropologie des Épidémies Émergentes (RAEE) est issu de l'extension thématique en 2016 du « Réseau SHS Ébola » (Sciences Humaines et Sociales Ébola) [4, 8, 18]. Ce premier réseau avait été créé par des chercheurs en 2014, au moment où l’épidémie ouest-africaine d’Ébola, ayant dépassé les frontières de la Guinée, du Libéria et de la Sierra Leone, constituait une menace pour l'Afrique de l'Ouest. Le réseau a permis aux chercheurs d’échanger sur les recherches menées dans une dizaine de pays pour avoir une compréhension plus complète de l’épidémie et du risque, et de faire connaître leurs travaux aux acteurs de la réponse^1^. L'une des activités majeures de ce réseau a été d'organiser en 2015 le colloque en sciences sociales consacré à l’épidémie d’Ébola (le seul tenu en Afrique), qui a rassemblé à Dakar 150 participants en sciences sociales et santé publique^2^. En 2016, le réseau élargit son champ thématique notamment pour aborder de nouvelles épidémies (fièvre de Lassa au Bénin, peste à Madagascar), et développe des activités d'information sur les approches sociales, de soutien aux échanges entre chercheurs et intervenants en santé publique, d'appui aux recherches et à la production d'outils méthodologiques et de diffusion d'informations^3^.

À la fin des années 2010, les besoins pour des recherches en sciences sociales sont croissants du fait de la multiplication des épidémies déclarées et de la structuration des capacités de diagnostic et de réponse en Afrique (avec en première ligne le Bureau Afrique de l'OMS et l'Africa CDC). Le RAEE, d'autres réseaux et des équipes spécialisées notamment sur le VIH se rejoignent, sur l'initiative de T. Giles-Vernick à l'Institut Pasteur de Paris, pour créer le réseau Sonar-Global, qui vise à mobiliser les sciences sociales sur les menaces infectieuses (zoonoses et résistance aux antimicrobiens) sur trois continents (Afrique, Asie, Europe) [9]^4^. Les deux réseaux – RAEE centré sur l'Afrique de l'Ouest (non exclusivement pour permettre l'approche comparative) et francophone, et Sonar-Global plus vaste et anglophone – vont collaborer sur une de leurs priorités communes: renforcer les capacités de recherche et d'expertise sur les dimensions sociales (au sens large) des épidémies et pandémies. Le réseau Sonar-Global, qui rassemble 11 puis 17 équipes de recherche, coordonné par l'Institut Pasteur de Paris, a produit des outils permettant d'aborder sur des bases validées la vulnérabilité des populations et l'engagement des communautés dans divers contextes [12, 20]. Alors que la pertinence de l'expertise en sciences sociales est de plus en plus reconnue, les besoins en ressources humaines s’étendent pour des professionnels compétents en sciences sociales de la santé et informés des questions spécifiques aux épidémies et pandémies.

## Première étape du processus: développer des supports de formation innovants et accessibles avec Sonar-Global

Une des priorités du réseau Sonar-Global est d'accroître les capacités de formation spécifiquement sur les dimensions sociales des épidémies. Dès 2017, le RAEE s'investit dans le programme RIPOST qui vise à concevoir des modules pédagogiques et délivrer des formations en épidémiologie d'intervention et sur les approches anthropologiques en situation d'urgence auprès de 250 professionnels de santé de 6 pays d'Afrique de l'Ouest. Des modules pédagogiques et supports de cours (27 heures) sont élaborés pour des formations dont la première session fut assurée par Marc Egrot, Roch Houngnihin et Khoudia Sow à Ouidah en 2018. Le programme, mis en place par un consortium à la demande de l'OOAS (Organisation Ouest Africaine de la Santé), en appui à la création des IMC (Institut médicaux de coordination) nationaux fédérés dans le nouveau CDC Afrique (Africa Centres for Disease Control and Prevention) et son bureau Afrique de l'Ouest à Abuja, n'a pas été mené à son terme.

En 2019, une analyse des formations universitaires sur les menaces infectieuses dans l'espace universitaire européen montre que parmi les très nombreux cycles et diplômes consacrés aux épidémies et pandémies, une infime proportion aborde les dimensions sociales au-delà d'une introduction très courte. Deux équipes de chercheurs, basées à l'Amsterdam Institute for Global Health and Development (AIGHD, Pays-Bas) et au Centre régional de recherche et de formation de Fann à Dakar (CRCF, Sénégal), vont proposer des supports de formation spécifiques, susceptibles d’être utilisés indépendamment ou en complément de ces formations. En termes de format, la production de curriculums, conformes aux approches didactiques des dispositifs universitaires des pays anglophones, devrait faciliter l'intégration dans les programmes existants. En termes de cibles et d'objectifs, deux types de supports semblent nécessaires aux anthropologues et médecins membres du réseau: pour les professionnels des sciences sociales, un document de spécialisation sur les épidémies et pandémies permettant, à partir de bases en sciences sociales de la santé, de développer des thématiques de recherche pertinentes et d'exercer une expertise en collaboration; pour les acteurs de la réponse, un outil d'initiation aux sciences sociales appliquées aux épidémies et pandémies permettant de saisir les problématiques sociales pertinentes et de préciser celles qui nécessitent des enquêtes pour ajuster les interventions en santé publique. Compte tenu de son ampleur, le champ thématique est subdivisé pour distinguer d'une part les épidémies émergentes, abordées de manière générale indépendamment de leur origine, et d'autre part la résistance aux antimicrobiens, qui soulève des questions spécifiques.

Le processus qui a permis de définir les contenus des curriculums de formation est basé à la fois sur l'expertise, l'expérience de formations antérieures^5^ et la contribution de chercheurs spécialisés en sciences sociales des épidémies et santé publique, ayant une expérience opérationnelle de recherche sur les épidémies et pandémies. Les outils concernant les épidémies émergentes ont été élaborés par l’équipe basée au CRCF (Anthony Billaud, Alice Desclaux, Khoudia Sow) en collaboration avec des chercheurs membres de Sonar-Global et du RAEE et ont été en partie inspirés des modules RIPOST, alors que les outils concernant la résistance aux antimicrobiens étaient conçus par l’équipe basée à l'AIGHD (coordonnée par Danny de Vries). À Dakar, le travail d’élaboration par l’équipe a alterné avec la consultation d'autres experts (socioanthropologues en poste dans des universités, instituts de recherche et institutions internationales, ingénieur pédagogique, historien et philosophe, médecins de santé publique) puis a été validé par un comité scientifique international composé de chercheurs en sciences sociales spécialisés sur les épidémies du Nord et du Sud^6^.

Un premier atelier organisé dans le cadre de Sonar-Global a eu lieu en novembre 2019 pour définir le format de l'outil didactique, sélectionner les thématiques clés et les contenus scientifiques (notions théoriques, références bibliographiques, études de cas et résultats de recherche). Pendant la pandémie en 2020, les échanges sur la base proposée en conclusion du premier atelier ont eu lieu de manière distante; ils ont permis de produire une première version du curriculum en français, composée de 13 chapitres correspondant à autant de modules de formation. Certains de ces modules ont été utilisés par les membres du groupe au cours de leurs activités d'enseignement. Un second atelier a eu lieu en novembre 2021 pour tirer parti de ces expériences et tester l'ensemble de la formation en présentiel, sur une version actualisée intégrant des éléments relatifs à la pandémie de SARS-CoV-2, avec une équipe composée en partie des chercheurs qui avaient participé au premier atelier et en partie de nouveaux chercheurs, notamment des juniors, afin de pouvoir poser un nouveau regard sur le processus et les contenus. Dans les suites de cet atelier, la version définitive du curriculum a été examinée et validée par une autre équipe de Sonar-Global, finalisée, traduite en anglais et postée sur le site Internet avec le curriculum destiné aux acteurs de santé, et les deux curriculums (l'un pour les professionnels des sciences sociales, l'autre pour les acteurs de la réponse) portant sur les dimensions sociales de la résistance aux antimicrobiens. Enfin, alors que le dernier atelier avait permis d'identifier la nécessité de disposer d'un document en français davantage développé, l’équipe du CRCF a rédigé un manuel avec l'appui des membres du RAEE sur la base du curriculum, qui a été complété et actualisé puis publié fin 2022 [5]^7^.

## Deuxième étape du processus: former des chercheurs et acteurs avec le RAEE

La première session de la « formation de formateurs » RAEE, destinée à des professionnels des sciences sociales, a eu lieu en novembre 2022 (Tableau I). L'objectif était de former une première génération de formateurs ouest-africains qui pourraient par la suite intégrer tout ou partie de cette formation dans leurs activités universitaires. La session a été organisée par le CRCF et l'IRD (TransVIHMI, LPED), avec le soutien financier de l'Agence nationale de recherche sur le Sida et les maladies infectieuses émergentes (ANRS MIE) et du RAEE devenu Groupement de recherche international Sud (GDRI-Sud) avec le soutien de l'IRD^8^.

**Tableau I:** Caractéristiques principales de la formation internationale de formateurs RAEE 2022 Main characteristics of 2022 international course for RAEE trainers

Format global: formation sur une semaine (28 heures, 5 jours) en présentiel ou distanciel, complétée par des lectures personnelles de documents mis à disposition (manuel, références bibliographiques) Profil des apprenants: universitaires en sciences sociales de niveau master et doctorat (20), professionnels en santé publique et santé communautaire (2), socio-anthropologues en institutions ou ONG de santé (2), équipe d'encadrement (5) Format pédagogique: Approche inversée. Chaque cours/module est élaboré à l'avance par deux participants (junior et senior) sur la base du manuel et des références bibliographiques. Chaque participant doit préparer deux cours et présenter un de ces deux cours sur l'ensemble de la semaine. Les présentations sont suivies de temps de questions et discussions. Des sessions spécifiques quotidiennes sont destinées à discuter le format et les contenus scientifiques. Disciplines des encadrants: anthropologie, santé publique et communautaire avec compétences en recherche multidisciplinaire et opérationnelle. Contenu didactique: notions théoriques et appliquées concernant les dimensions sociales de la maladie, études en sciences sociales pour la santé publique, à propos de diverses épidémies (Sida, Ébola, grippes, SRAS, MERS, SARS-CoV-2, antibiorésistance) sur la base des modules du curriculum et du manuel. Compléments didactiques: programme, glossaire, grilles d’évaluation, indications didactiques extraits du curriculum et du manuel.

L’évaluation de la formation par les participants et les encadrants, au vu des principes mobilisés pour la définir et des objectifs pédagogiques, a montré sa pertinence, son utilité et ses capacités à être adaptée à divers contextes de transmission de connaissances et appropriée par les chercheurs. Certains participants ont mis en avant l'intérêt qu'a représenté pour eux le temps de recul sur les pratiques et l'acquisition de capacités d'analyse à propos de questions auxquelles ils avaient été confrontés sur le terrain sans pouvoir y répondre. D'autres ont insisté sur la richesse des sujets traités, parfois éloignés de leur expérience personnelle, mais qu'ils sont maintenant en mesure d'aborder. Enfin, l'ensemble des participants ont apprécié les échanges et le travail collectif, en avançant que les épidémies, faits sociaux engageant la complexité, nécessitent de disposer de contacts avec d'autres chercheurs pour mobiliser des expertises parfois focalisées sur des thèmes précis, et pour échanger collectivement sur les approches les plus actualisées et pertinentes face à une situation nouvelle.

Cette première utilisation avec des participants déjà experts pour une partie d'entre eux, à la fois enthousiastes à propos de la mise à disposition d'un outil pédagogique servant de base pour construire un cours et capables de critiques sur les formats et contenus au vu de leur expérience, a permis la validation du modèle.

Des sessions de formation de plus courte durée ont ensuite été réalisées, notamment au Burkina Faso et au Sénégal:

- Au Burkina Faso, la formation a été dispensée auprès d'enseignants-chercheurs et chercheurs en sciences sociales, avec la coordination et l'encadrement de Blandine Bila à l'Institut de recherche en sciences de la santé (IRSS, Ouagadougou). Cette formation comportait deux volets. Le volet préparatoire consistait pour les apprenants à relire les cours dans le draft du manuel, et à les amender dans la forme, le contenu (thèmes, méthode, apport des références bibliographiques locales…), avant de les discuter en plénière pendant le cours qui a duré trois jours, pour les compléter et les adapter au contexte national. Les modules du programme de formation ont été ainsi présentés par les participants (répartis par deux pour chaque module) à raison de quatre par jour pendant les deux premiers jours du cours et cinq le dernier jour. Le rapport de formation comportant les suggestions, questionnements et amendements issus de la session a été transmis à l’équipe de rédaction inter-pays.
- Au Sénégal, la formation a été réalisée auprès d'acteurs de santé publique, santé communautaire, intervenants sociaux mobilisés avec l'appui du Conseil national de lutte contre le Sida, avec la coordination et l'encadrement de Khoudia Sow au CRCF. Cette session s'est appuyée sur le curriculum destiné aux professionnels de santé et acteurs de la réponse, qui partage des éléments communs avec le premier curriculum. Le format a été différent de la formation réalisée au Burkina Faso: les thèmes des sessions ont été définis dans une approche davantage opérationnelle; une sélection plus étroite des modules a été effectuée suivant les priorités du public et sans volonté d'aborder l'ensemble des thèmes; les notions théoriques discutées ont été plus limitées et les exemples plus fournis; l'approche a privilégié la mise en situation et la résolution de problèmes. Le manuel a fourni les connaissances pratiques et théoriques nécessaires en sciences sociales pour discuter les situations, le glossaire indispensable pour établir des échanges sur des bases rigoureuses, et les études de cas qui ont été discutées. Des intervenants opérationnels aux profils divers (médecins, assistants sociaux, infirmiers qui ont été en charge de la riposte des épidémies de VIH, Ébola et Covid à différents niveaux du système de santé) ont partagé leurs expériences et leurs points de vue sur les dispositifs de préparation et de riposte, les effets de ces dispositifs, les stratégies d'adaptation développées au cours des épidémies, ainsi que sur les problématiques sociales telles qu'ils les ont vécues.

Ces deux expériences ont permis de valider la méthode de formation et les documents (manuel, références bibliographiques), et la possibilité de les adapter d'une part à des publics différents et d'autre part à des contextes nationaux distincts. Cette adaptation repose sur le choix de l'approche, des méthodes didactiques, et sur la sélection des intervenants et des études de cas.

## Les connaissances à transmettre sur les dimensions sociales des épidémies

La définition des contenus devait être compatible avec les contraintes d'une transmission de connaissances « suffisantes et nécessaires » pour appréhender les questions clés en lien avec une épidémie. La durée de la formation est limitée pour qu'elle puisse être intégrée *in fine* dans des cursus universitaires diplômants^9^. L'approche a consisté à sélectionner les notions théoriques et les études de cas les plus pertinentes, dans une optique de santé publique (connaissance pour l'intervention) et de sciences sociales (connaissance pour la compréhension et l'approfondissement des savoirs et modèles d'interprétation).

En l'absence de formation préexistante en anthropologie de la santé pour nombre de chercheurs en sciences sociales (de niveau Master et plus) qui doivent être mobilisés, il est apparu nécessaire de transmettre aux participants des savoirs et des savoir-faire, dans les domaines suivants^10^:

- En matière de savoirs: des bases théoriques en anthropologie médicale (incluant la présentation de concepts clés pouvant être aisément mobilisés et la discussion critique de concepts véhiculaires flous, ambigus ou culturalistes tels que « communautés »); des éléments d'histoire globale des épidémies complétés par des connaissances (biocliniques, en santé publique, épidémiologiques, sociales) à propos d’épidémies spécifiques, suscitant la réflexion par la comparaison et la mise en perspective de contextes et dynamiques épidémiques variés; des notions de base en épidémiologie et santé publique (tels que: indicateurs épidémiologiques, critères d’évaluation des interventions et services) pouvant être utilisées dans une approche interdisciplinaire; des études et recherches en sciences sociales de la santé appliquées à la lutte contre les épidémies.
- En matière de savoir-faire: des méthodes de recherche et gestion de ressources documentaires en anthropologie médicale; des démarches de sélection des méthodes d'enquête, de recherche et d'intervention en sciences sociales de la santé (incluant les apports et limites des méthodes rapides et des méthodes participatives); les approches de la vulnérabilité sociale et de l'engagement communautaire; les spécificités (éthiques et méthodologiques) des enquêtes qualitatives autour des maladies infectieuses; la définition commune (par des acteurs des deux champs disciplinaires) d’études en sciences sociales et santé publique.

Le Tableau II présente les thématiques et contenus de la formation, actualisés et complétés après la pandémie de Covid.

**Tableau II:** Thématiques et contenus pédagogiques de la formation en Anthropologie appliquée aux épidémies émergentes Themes and pedagogical contents of the course on anthropology applied to emerging epidemics

Module	Contenus pédagogiques	
	Notions théoriques	Résultats de recherche Études de cas (sélection)
1. Introduction	Présentation des notions clés sur les épidémies en sciences sociales et en sciences de la santé. Comprendre la multiplication des maladies infectieuses émergentes. Crise épidémique et régime dexception. Rapport global/local dans la réponse. Besoins en recherche et expertise. Construction pluridisciplinaire des connaissances, entre savoirs et pouvoirs.	Les facteurs écologiques des maladies infectieuses émergentes. Les influences pérennes des logiques politiques dans la production des connaissances sur les épidémies: le cas du choléra.
2. Le cadre de la réponse aux épidémies	Le dispositif de santé globale: institutions et acteurs, instruments de la réponse. Notions d’épidémiologie et santé publique. Diverses approches en sciences sociales de la réponse aux épidémies, entre biomédecine et culture, systèmes de soin et systèmes de sens.	Le mandat de l'OMS face à une épidémie. Le rôle des ONG internationales dans la réponse aux épidémies.
3. Émergence et One Health (Une seule santé)	Les relations humains-animaux dans les infections zoonotiques. Environnement et santé planétaire. L'approche One Health, modèle de compréhension et de collaboration. Savoirs locaux et approches anthropologiques de la santé multi-spécifique.	L'investigation du *spill over* entre chauves-souris et enfant pour Ébola en 2013. Relations inter-espèces et épidémie de rage humaine en Inde.
4. La résistance aux antimicrobiens (RAM)	La RAM dans une perspective médicale et de santé publique. Les facteurs microsociaux et systémiques. Les approches en sciences sociales des anti-infectieux, du mésusage à la vie sociale des médicaments. Accompagner les stratégies de lutte par les sciences sociales.	Vivre avec le SARM, une superbactérie à l'origine d’épidémies nosocomiales dans la population. Une stratégie de lutte: la campagne AWARE de l'OMS .
5. Le risque infectieux. Inégalités, stigmatisation et prévention	La notion de risque infectieux, les dimensions objectivées du risque et les inégalités dexposition de niveaux global et local. Les dimensions subjectives et socialisées du risque au travers de ses représentations et de la stigmatisation, et son contrôle en santé publique. La communication sur le risque et la prévention vues par l'anthropologie.	La priorisation des risques par des individus ou des populations. La co-construction des messages avec les populations.
6. Les savoirs. Circulations et interprétations	La construction sociale des connaissances et des représentations sociales autour des épidémies. L'infodémie, les infox et la circulation de l'information. Les interprétations des épidémies, théories causales, discours néo-traditionnels et religieux. Les dimensions politiques et la mobilisation des récits épidémiques dans les réponses.	L'origine de la maladie à virus Ébola vue par les jeunes du Burkina Faso. La prévention d’Ébola en contexte de pèlerinage religieux.
7. Les services de santé. Lieux de risque et de soins	Les soins et la prévention des infections dans les services de santé. Le triage et les unités spécialisées. Les soins en contexte d'incertitude. L'exposition des acteurs de santé à divers risques (infectieux, sociaux, psychiques) et leur vulnérabilité. L'impact des règles de biosécurité sur les relations soignants-soignés. L'impact de l’épidémie sur les soins d'autres maladies.	Les agents de sécurité hospitaliers en première ligne face au risque infectieux. Les relations soignants-soignés dans un service hospitalier au temps du Covid en France.
8. Les mesures de santé publique. Enfermement et distanciation	Diversité et évolutions des « mesures de santé publique »: quarantaine, isolement, distanciation sociale, fermeture et contrôle des frontières. Les dimensions sociopolitiques de la distanciation. Interprétations dans divers contextes épidémiques. Effets des mesures sur les rapports sociaux, impact économique et enjeux politiques.	Repères historiques à propos de la quarantaine et ses failles. Les mesures aux frontières de la Côte d'Ivoire pendant l’épidémie d’Ébola: entre légitimité politique et impératifs socioculturels.
9. Les expériences. Souffrance des malades et mobilisations	Maladie vécue, socialisée et itinéraires de soins en temps d’épidémie. Perceptions, statuts de survivants et de patients experts. Les mobilisations associatives et les modèles de « lengagement communautaire »: critique de la notion.	Vouloir vivre ou se laisser mourir: deux expériences de la maladie. Être identifié comme cas alerte ou suspect: l'expérience d’Ébola.
10. La mort. Sens et rituels	Les pratiques de sécurisation des corps et les rites funéraires. Les formes de gestion du risque lié à la prise en charge des personnes mourantes et décédées. Les conditions pour articuler impératifs de biosécurité et impératifs culturels. Assurer pendant les crises épidémiques une mort considérée comme digne.	Rituel funéraire par vidéoconférence pendant la pandémie de Covid. Les morts inacceptables en temps de Covid: les témoignages de soignants en Italie.
11. La vaccination. Innovation, équité et hésitation	L'histoire et les fondements médicaux de l'utilisation des vaccins. L'acceptabilité et la demande: déterminants structurels et conjoncturels. Les notions d’équité et d'hésitation vaccinale dans le contexte de la santé globale. Aspects sociaux des épidémies induites par le déficit de vaccination.	Le contexte du boycott de la vaccination contre la polio au nord du Nigéria en 2003. Les informations et perceptions de la vaccination au Sénégal en période de Covid.
12. Les cycles épidémiques. Préparation, réponse et rétablissement	Le cycle épidémique et le cadre opérationnel. La préparation, la réponse et le rétablissement post-crise aux niveaux global et local. La surveillance des épidémies et ses enjeux sociaux. L'imaginaire de la préparation. Les approches de ces trois phases, en particulier pour la recherche.	Les enjeux sociaux de la surveillance à base communautaire en Guinée. Imaginaires de la maladie X^1^
13. Défis, méthodes, éthique et gouvernance	Les méthodes et enjeux éthiques de l'enquête pendant la crise épidémique. Les défis de l'interdisciplinarité et de l'application « dans l'urgence ». Les contributions des chercheurs et les dimensions éthiques de l'enquête. Des méthodes adaptées. L'articulation entre approches des sciences sociales et réponses en santé publique (partage des résultats).	Évaluation globale de la vulnérabilité et de l'engagement communautaire pendant la pandémie de Covid. Plateformes web proposant des documents en sciences sociales sur les épidémies.
Conclusion	Perspectives pour aborder les épidémies émergentes avec un éclairage anthropologique dans le « monde d'après » la pandémie de Covid.	

1 Le terme « Disease X » a été adopté par l'OMS et les instances de santé globale pour désigner une prochaine épidémie ou pandémie hypothétique (dont l'agent pathogène nest pas connu a priori) à des fins de prédiction et de préparation.

Les contenus incluent des analyses et exemples concernant principalement l’épidémie de Sida (pathologie la plus étudiée en sciences sociales et santé publique), l’épidémie d’Ébola en Afrique de l'Ouest (archétype des maladies infectieuses émergentes) et la pandémie de Covid (qui a suscité d'importantes avancées en particulier sur les plans analytique et méthodologique), et secondairement une variété d'autres épidémies.

## Conclusion

Le RAEE a mis en place un processus de renforcement des capacités des professionnels des sciences sociales sur les épidémies, dans un objectif de « préparation » à la prise en compte des dimensions sociales des épidémies émergentes et ré-émergentes. La démarche est basée sur les connaissances théoriques et les expertises empiriques multidisciplinaires de spécialistes en sciences sociales et biomédicaux acquises dans le contexte des épidémies de VIH, Ébola et Covid. Des outils didactiques ont été développés, testés, validés, et déclinés sous plusieurs formats (curriculums, manuel). Une formation de formateurs a été assurée, qui a été suivie d'une dissémination des supports et du modèle de la formation. Cette expérience, complétée par les évaluations des participants, met en lumière ses points forts: les apports de connaissance sur des épidémies dont les aspects épidémiologiques et de santé publique étaient peu connus des participants; l'approche systématique qui permet d'aborder des dimensions sociales parfois peu visibles sur le terrain malgré leur importance; les enseignements fondés sur des données scientifiques actualisées et prenant en compte des résultats de recherche récents sur la pandémie de Covid; les apports méthodologiques et éthiques, complémentaires aux enseignements universitaires usuels, surtout pour la partie opérationnelle; la disponibilité d'un manuel, besoin qui avait été mis en exergue par l’évaluation de la formation MIAA (Maladies Infectieuses et Anthropologie en Afrique) réalisée en Guinée en 2017 [6].

Un des partis pris du RAEE (ainsi que de Sonar-Global) a été de favoriser l'accessibilité des documents didactiques et de la formation en les diffusant largement, et en encourageant leur utilisation sous licence C-C-C, qui implique un droit à la réutilisation et à la transformation, mais à la condition que les auteurs initiaux soient cités et que les versions suivantes ne soient pas commercialisées, ces deux clauses s'appliquant également aux versions ultérieures. Ceci devrait permettre de multiplier les initiatives de formation, notamment par des binômes associant enseignantschercheurs en sciences sociales et en santé publique, auprès des nombreuses catégories de professionnels de santé (humaine et animale). Les comités nationaux One Health et les centres opérationnels traitant les urgences sanitaires bénéficieraient en priorité de ce type de formation pour mieux intégrer les sciences sociales dans leurs approches et leurs activités. La formation fait partie de la stratégie de préparation aux épidémies et pandémies, mais n'est pas exclusive: une préparation plus complète inclurait l'identification des connaissances existantes en socio-anthropologie sur les populations, la santé et la maladie dans les espaces concernés, par exemple en Afrique de l'Ouest. Il serait aussi indispensable d'identifier les questions de recherche et thématiques prioritaires à développer et adapter pour diverses menaces infectieuses. Des compétences complémentaires spécialisées pourraient être mobilisées, notamment en sciences de la communication. Enfin, la production de nouvelles connaissances devrait être soutenue dans la phase de préparation au travers de collaborations pluridisciplinaires, ainsi que des dispositifs d’échanges entre les chercheurs et les différents profils d'acteurs de la réponse aux niveaux micro- et macrosocial, pour prétendre à être préparé à faire face aux dimensions sociales de nouvelles épidémies.

## Contribution des auteurs

AD: conception et mise en œuvre du dispositif, rédaction et correction du manuscrit. BB, ME, KS: conception et mise en œuvre du dispositif, discussion et correction du manuscrit.

## Liens d'intérêts

Les auteurs ne déclarent aucun lien d'intérêts.
